# Medication Experiences

**DOI:** 10.3390/pharmacy9020079

**Published:** 2021-04-13

**Authors:** Jon C. Schommer

**Affiliations:** College of Pharmacy—Twin Cities, University of Minnesota, 308 Harvard Street, SE, Minneapolis, MN 55455, USA; schom010@umn.edu; Tel.: +1-612-626-9915; Fax: +1-612-625-9931

Welcome to the “Medication Experiences” Special Issue in the journal—Pharmacy—an open access journal focused on pharmacy education and practice. In February 2020, an invitation was dispersed to scholars in the medication experiences domain, asking them to submit a manuscript to this Special Issue no later than 30 November 2020. We invited these colleagues to think about a full breadth of topics including, but not limited to: (1) individuals’ subjective lived experience of taking medications in their daily lives, (2) symbolism that medications hold for people, (3) meanings of medications for people (4) positive or negative bodily effects people experience from medications, (5) dealing with the unremitting nature of chronic medication use, (6) exerting control over their medications, (7) medications as a life savior or a life burden, (8) beliefs, information processing and decision making about medications, (9) helpful service designs for using medications, and (10) patient-reported outcomes regarding medications.

The overall goal of this Special Issue on “Medications Experiences” is to give the reader a state-of-the-art synopsis of the medication experience domain at this point in time. To accomplish this goal, we sought papers that address the social, psychosocial, political, legal, historic, clinical, and economic factors that are associated with medication experiences. Papers that translate concepts from other domains into the medication experiences realm were welcome for this Special Issue. We sought manuscripts of all types including (1) reviews, (2) commentaries, (3) idea papers, (4) case studies, (5) demonstration studies, and (6) research studies.

With this foundational context described and the invitations sent, we waited to learn about what would be submitted in a timeframe of just several months. We are pleased to report that 20 articles have been published in this Special Issue and represent the work of over 50 scholars in this domain. To receive such a response from busy colleagues in such a short time-frame is incredible. As we review the articles in this Special Issue, a great deal can be learned about (1) conceptual foundations for the medication experience, (2) understanding the experiences of unique patient populations, and (3) application of the concept through tailored services.

## 1. Conceptual Foundations

The “Medication Experience” concept continues to develop, and several manuscripts provide profound insights. Nascimento and colleagues provide a commentary using the phenomenology of Merleau-Ponty as an important theoretical framework and describe resolution, adversity, ambiguity, and irrelevance as components of the medication experience. Further, they describe multiple pathways over time, cyclical structures, the role of habit and routine, and the importance of existential feelings for understanding this concept. Hillman and colleagues report findings from a conceptual analysis of existing literature that are consistent with Nascimento’s work showing aspects of ambivalence, vulnerability, social construction, pragmatism, contextual framing, nuance, and the ongoing nature of the concept. The article by Glinert applies ethnographic and social semiotic models of discourse and communication and reveals the roles of the actors, messages, mediums, genres, and contextual factors on the medication experience.

Langley applies health economics to the medication experience concept and reveals how comparative effectiveness decisions related to medications must transition from the current standards to those that embrace a commitment to disease-specific, patient-centered evidence. Oliveira and colleagues apply the medication experience concept to a comprehensive medication review and propose courses of action that pharmacists can take to help patients overcome barriers or change therapy to match patients’ unique characteristics. Isetts and colleagues propose ideas regarding patient-centered care within the medication experience and reveal how patient-specific preferences will lead to varying therapeutic relationships between providers and patients.

These five articles will be of great interest to readers who are interested in thinking about the foundations of the “Medication Experience” concept. The authors provide keen insights and also provide provocative ideas that warrant further research.

## 2. Unique Patient Populations

Another emerging theme for medication experiences described in this Special Issue is to view them through the existential meanings that unique patient populations have. Ibrahim and colleagues describe experiences, beliefs, and behaviors for patients with chronic disease in both the United States and Oman. Flood and colleagues focus on people with intellectual disabilities, while Gorsen and colleagues describe perspectives of caregivers and health care providers for infants with reflux. Shah and colleagues focus on over-the-counter medications. Two articles by Cernasev and colleagues focus on African-born persons living with HIV in the United States. An article by Hall and two by Cernasev and colleagues focus on opioid use. Compagner and colleagues focus on medications used to treat depression. These 10 articles reveal how unique and specialized medication experiences are depending upon the context in which medications are used.

## 3. Tailored Services

Finally, three articles in this Special Issue reveal how services can be tailored to help meet patients’ needs. Phi and colleagues describe a medication packaging service to help medication adherence behaviors. Witry and colleagues describe a medication experience questionnaire that can be administered at home and used by pharmacists to tailor services. Hartley and colleagues describe a telephonic service to help patients with uncontrolled asthma.

We trust that you will find the articles in this Special Issue not only informative but inspiring as well. We greatly appreciate the colleagues who were willing to share their work with us. We also wish to thank the editorial staff who coordinated the review and publishing processes. Their professionalism is highly valued. Finally, we wish to thank you, the reader. Please apply the ideas in these articles to your work. Expand upon them. Challenge them. Then, share your work with us.

We would welcome your submission to another Special Issue called “Medication Experiences II.” Submissions for that Special Issue are due by 30 November 2021. The journal has agreed to publish papers for this Special Issue free of charge. The typical article processing charge is 1400 CHF (Swiss Francs), so please correspond with Christina Zhang, Assistant Editor (christina.zhang@mdpi.com), for setting up the charge waiver. If more than 10 papers are published, a book edition will be prepared. More information may be found at https://www.mdpi.com/journal/pharmacy/special_issues/medication_experiences_II (accessed on 25 March 2021).

## Data Availability

Not applicable.

